# A Review of Robotics in Neurorehabilitation: Towards an Automated Process for Upper Limb

**DOI:** 10.1155/2018/9758939

**Published:** 2018-04-01

**Authors:** E. D. Oña, R. Cano-de la Cuerda, P. Sánchez-Herrera, C. Balaguer, A. Jardón

**Affiliations:** ^1^Robotics Lab, Department of Systems Engineering and Automation, University Carlos III of Madrid, Avda. de la Universidad 30, 28911 Leganés, Madrid, Spain; ^2^Department of Physical Therapy, Occupational Therapy, Physical Medicine and Rehabilitation, University King Juan Carlos, Avda. de Atenas s/n, 28922 Alcorcón, Madrid, Spain

## Abstract

Robot-mediated neurorehabilitation is a growing field that seeks to incorporate advances in robotics combined with neuroscience and rehabilitation to define new methods for treating problems related with neurological diseases. In this paper, a systematic literature review is conducted to identify the contribution of robotics for upper limb neurorehabilitation, highlighting its relation with the rehabilitation cycle, and to clarify the prospective research directions in the development of more autonomous rehabilitation processes. With this aim, first, a study and definition of a general rehabilitation process are made, and then, it is particularized for the case of neurorehabilitation, identifying the components involved in the cycle and their degree of interaction between them. Next, this generic process is compared with the current literature in robotics focused on upper limb treatment, analyzing which components of this rehabilitation cycle are being investigated. Finally, the challenges and opportunities to obtain more autonomous rehabilitation processes are discussed. In addition, based on this study, a series of technical requirements that should be taken into account when designing and implementing autonomous robotic systems for rehabilitation is presented and discussed.

## 1. Introduction

According to the findings obtained in the context of a Global Initiative on Neurology and Public Health carried out by the World Health Organization (WHO), many of the neurological disorders are chronic and progressive, constitute a global public health problem [1], and affect especially the elderly people. In addition, a higher life expectancy makes the population of people over 60 increasingly higher [2]. The main patient groups served by the rehabilitation service in the United Kingdom are for neurological pathologies, as a survey reported [3]. 70% of respondents provided neurological rehabilitation services for people with stroke, multiple sclerosis, traumatic brain injury, degenerative neurological diseases, and other neuromuscular conditions. Other services that were represented were those that provided rehabilitation to people with severe single-incident brain injury (10%), spinal injury (9%), amputees (5%), musculoskeletal disability (4%), learning disabilities (1%), and pain (1%). In Spain, a similar situation is detected where musculoskeletal and articular disability (50%), neurological diseases (15%), traumatic injuries (29%), and others (6%) were treated in the rehabilitation services [4].

This situation, together with the need for rehabilitation and assistance for people with disabilities, means that robotic care and rehabilitation may play an important role in the years ahead.

Nowadays, research on the use of robotic systems in different fields related to healthcare is widespread [5–7]. In the field of rehabilitation, scientific literature shows various classifications of such systems according to their level of interaction [8], the extremities that are treated [9–12], the modularity of the rehabilitation robots [13, 14], control strategies [15, 16], and the effectiveness of treatment [17–20]. However, no analysis has been done of the rehabilitation process as such, and the contribution of robotics in the different stages of the rehabilitation cycle or process has not been studied.

In this paper, a systematic literature review is conducted to identify the contribution of robotics for upper limb neurorehabilitation highlighting its relation with the rehabilitation cycle and to clarify the prospective research directions in the development of an autonomous rehabilitation process.

## 2. The Rehabilitation Process

The World Report on Disability by the WHO and World Bank [21] provides a definition of rehabilitation: “a set of measures that assist individuals who experience, or are likely to experience, disability to achieve and maintain optimal functioning in interaction with their environments.”

Despite this, the term rehabilitation covers a wide field of applications, being a subject to different connotations in a world characterized by a profound cultural diversity. Meyer et al. [22] provided a conceptual description of rehabilitation: “it is the health strategy which is based on the WHO's integrative model of functioning, disability, and health, with the goal to enable persons with health conditions experiencing or likely to experience disability to achieve and to maintain optimal functioning in interaction with the environment.”

**Figure 1:**
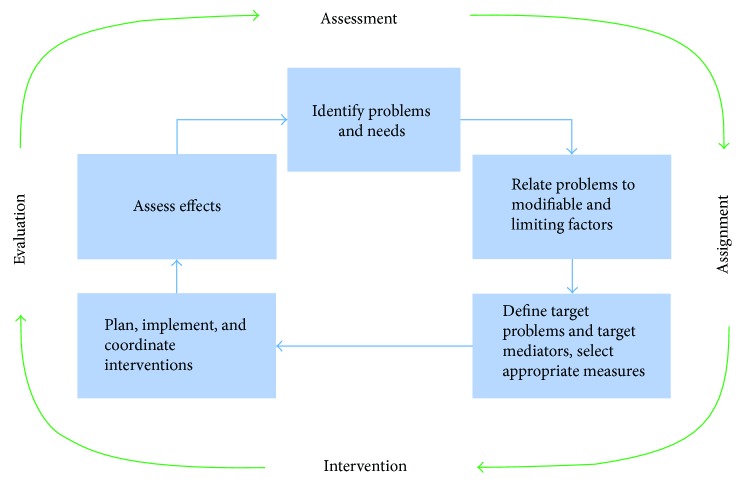
The rehabilitation cycle [21].

The health strategies can be different, but they can share a series of steps to improve the patient's health status throughout the rehabilitation process. This process involves the identification of a person's problems and needs, relating the problems to relevant factors of the person and the environment, defining rehabilitation goals, planning and implementing the measures, and assessing the effects [21]. This approach is named the rehabilitation cycle (see Figure 1), which is taken from the World Report on Disability [21], and it was previously stated by Stucki and Sangha [23] and modified by Steiner et al. [24].

In a simplified way, the rehabilitation cycle includes four steps: assessment, assignment, intervention, and evaluation. The process takes place on two levels: the first corresponds to the guidance provided along the continuum of care and the second refers to the provision of a specific service [25].

From the point of view of the care guide, the assessment consists of the identification of the problems and needs of the person, the analysis of rehabilitation potential and prognosis, the definition of the long-term service, and the goals of the intervention program. Assignment refers to the inclusion of the person in a program of intervention in the most appropriate service for the treatment of their needs. For the guidance perspective, no specifications appear in the intervention. Evaluation refers to the service and the achievement of the intervention goal.

From the perspective of providing a specific service, the assessment includes the identification of the problems, the review and potential modification of the service or goals of the intervention program, the definition of the first goals of the rehabilitation cycle, and the objectives of the intervention. The assignment step refers to the allocation of professionals and health interventions necessary to achieve the intervention objectives. The intervention consists in the specification of the techniques, measures, and the definition of target values that must be achieved within a predetermined period of time. Finally, the evaluation determines the achievements of the objectives with respect to the specific indicators, the goals of the rehabilitation cycle, and, ultimately, the goals of the intervention program. It also includes the decision regarding the need for another intervention cycle based on a new assessment.

### 2.1. The Rehabilitation Team

Rehabilitation requires the services of multiple healthcare providers who possess unique skills, training, and expertise that are employed for the full restoration of the patients' function and their optimal reintegration into all aspects of life [26]. Rehabilitation professionals have recently favoured the concept of “patient-centred therapy.” This is not meant to trivialize the patient's needs but rather to emphasize the patient as the director and arbiter of the interventions according to the patient's own desires [27].

The integration of the different medical means can be done through three working models [26, 28]: *(a) multidisciplinary team model*—in which team members interact and communicate among themselves, knowing the work of all the components and offering an evaluation and parallel but independent work; *(b) interdisciplinary team model*—where the team members share a formal space in which information is exposed (designed to facilitate the flow of lateral communication) and decisions are made around one or several common objectives (in this way, the treatments performed by the different professionals are not independent); and *(c) transdisciplinary team model*—which not only promotes communication among group members but also acquires knowledge from other related disciplines and incorporates them into the practice [29].

Because the interdisciplinary model is designed to facilitate lateral communication, it is theoretically better suited for rehabilitation teams [28].

### 2.2. Rehabilitation Measures and Outcomes

Rehabilitation measures are a set of recovery actions that target body functions and structures, activities and participation, environmental factors, and personal factors.

Rehabilitation outcomes are the benefits and changes in the functioning of an individual over time that are attributable to a single measure or set of measures [30]. These outcomes can be evaluated by the three main dimensions of the International Classification of Functioning, Disability and Health (ICF) [31]: body functions and structures, activities, and participation.

## 3. Neurological Rehabilitation

A particular case of rehabilitation is aimed at treating the problems caused by disorders affecting the nervous and neuromuscular system, known as neurorehabilitation. These types of disorders can produce mental or physical disabilities or both and are chronic and/or progressive.

Neurological rehabilitation can be defined as a process that aims to optimize a person's participation in society and sense of well-being. This definition highlights several important features: rehabilitation is not a particular type of intervention; the focus is on the patient as a person; the goals relate to social functioning, as well as health or well-being; and it is not a process restricted to patients who may recover, partially or completely, but applies to all patients left with long-term problems [32]. This will act on the deficiency, the limitation of activity, and the restriction of participation, constituting a holistic therapeutic approach [33].

The complexity of the problems caused by a neurological damage highlights even more the need for a team to work on its treatment, the interdisciplinary model being the most used [34]. The composition of the interdisciplinary team in neurorehabilitation is not completely defined, but there is a consensus on the basic members who should constitute the team. According to the Union of European Medical Specialists (UEMS), the interdisciplinary team must include the following medical professionals: physical therapist, rehabilitation nurses, rehabilitation physicians, occupational therapists, speech-language pathologist, psychologists, social workers, orthopaedics, and nutritionists [35].

The rehabilitation cycle shown in Figure 1 applies to the case of neurological rehabilitation with some nuances that are discussed below.

### 3.1. Assessment

The rehabilitation process starts with collecting data from the patient and others to establish: the problems; the causes of, and factors influencing, each problem; and the wishes and expectations of all interested parties. It is also important to consider the prognosis based on the diagnosis, natural history, distribution, and severity and type of the impairment, as well as other personal, social, and environmental factors [36].

To this end, a series of objective scales have been developed to assess the level of independence of patients. The three main domains of the ICF can be used with this aim as a clinical tool [37, 38]:

- Impairments: the typical body functions that need to be assessed in the neurological patient are those related to the functions of the joints, muscles, movements, and sensation and cognitive functions. Thus, some constructs of relevance are muscle, ranges of movement, attention, memory, and balance. There are scales classically encompassed at this level such as Beck Depression Inventory, Behavioral Inattention Test, Canadian Neurological Scale, Clock Drawing Test, Frenchay Aphasia Screening Test, Fugl-Meyer Assessment of Motor Recovery after Stroke, General Health Questionnaire-28, Geriatric Depression Scale, Hospital Anxiety and Depression Scale, Mini-Mental State Examination, Modified Ashworth Scale, Montreal Cognitive Assessment, Motor-Free Visual Perception Test, National Institutes of Health Stroke Scale, and Orpington Prognostic Scale.
- Activity: when examining a patient's activities, the therapist will examine whether they can do not only the tasks but also the quality with which the task is performed. According to Lennon's study [39], one of the most used scales for measuring the independence in stroke rehabilitation was the Barthel Index, followed by the Rivermead Motor Assessment and Functional Independence Measuring. More than a quarter of therapists (28%) were using outcome tools that they had devised themselves, which had not been tested for reliability or validity. Other examples of scales at this level are the following: Action Research Arm Test, Berg Balance Scale, Box and Blocks Test, Chedoke-McMaster Stroke Assessment Scale, Clinical Outcome Variables, Functional Ambulation Categories, National Rehabilitation Reporting System, Frenchay Activities Index, Modified Rankin Handicap Scale, Motor Assessment Scale, Nine-Hole Peg Test, Rivermead Mobility Index, Timed “Up and Go” Test, and Wolf Motor Function Test.
- Participation: this a more complex concept than impairments and activities, but it is fundamental to understand the patients and their life and help with planning treatment. Physiotherapy assessment of participation therefore focuses on those activities or roles in which patients take part in, patients are hindered in, and patients wish to work on and which could be improved and will inevitably deteriorate. Common scales used are the following: Canadian Occupational Performance Measure, EuroQol Quality of Life Scale, London Handicap Scale, Medical Outcomes Study Short Form 36, Nottingham Health Profile, Reintegration to Normal Living Index, Stroke-Adapted Sickness Impact Profile, Stroke Impact Scale, and Stroke Specific Quality of Life Scale.

### 3.2. Planning of Treatment

According to the pathology, the rehabilitation team designs a specific plan based on the diagnosis (problems identification) and disability of the patient. It is necessary to identify clear objectives related to the functional problems. Rehabilitation objectives normally follow the SMART rule because they must be specific, measurable, achievable, relevant, and time-limited [32].

There are three key areas that the rehabilitation process is broken down: (1) approaches that reduce disability; (2) approaches designed to acquire new skills and strategies, which will maximize activity; and (3) approaches that help to alter the environment, both physical and social, so that a given disability carries with it minimal consequent handicap. The planning of a neurological rehabilitation program should consider the previous three approaches, in addition to the SMART rule.

### 3.3. Intervention: Specific Methods

Specific rehabilitation interventions include those related to physical medicine, occupational therapy, speech and language therapy, dysphagia management, neurophysiological interventions, psychological assessment and interventions, nutritional therapy, and other interventions [25]. A wide range of specific techniques is used in the practice of rehabilitation [40]. These techniques used to treat different patients vary considerably across different geographical locations.

At present, the evidence suggests that to be effective, rehabilitation requires the practice of activities in the most relevant possible environments, rather than undertaking analytical exercises aimed at changing impairments [41]. This is sometimes referred to as task-specific training. However, other approaches are known such as facilitation techniques (such as Bobath concept, Brunnstrom technique, Kabat method, or Rood method), modern techniques (such as treadmill training with body weight support, constraint-induced movement therapy, or functional electrical stimulation), or compensation techniques.

### 3.4. Evaluation

In this phase, the physical condition of the patient is reevaluated in order to determine the effectiveness of the treatment, based on the SMART objectives [32] initially raised. The considerations for discharge in the case of the neurological patient are very varied, since the clinician must determine whether the improvement achieved is sufficient from the medical point of view of the patient (patient-centred practice).

Previous quantitative investigations and case studies have shown that the use of patient-centred goal planning with adults undergoing neurological rehabilitation can improve self-perceived and observed goal performance and satisfaction [42]. A patient-centred approach involves goals that are set by the patient on the basis of his or her own definition of the problems. This approach enables greater self-determination and control and enhances the person's potential for active participation.

In addition, one must take into account the underlying pathological process, the chronic nature of certain pathologies, the need for supervision and/or the continuity in the absence of an expressive face-to-face rehabilitation treatment, or the degenerative and progressive character of some neurological pathologies, such as Parkinson's disease, multiple sclerosis, or Alzheimer's disease.

## 4. Robotics in Healthcare: Neurorehabilitation of Upper Limb

In this section, this review will highlight the particular aspects of the rehabilitation cycle applied to upper limb neurorehabilitation performed with the assistance of any kind of robotic system.

### 4.1. Material and Method

#### 4.1.1. Search Methods

The authors undertook a literature search in October 2017 about robot-assisted upper limb rehabilitation in neurological diseases, using keywords such as robot, neurological, rehabilitation, upper, limb, extremity, arm, hand, neurorehabilitation, intervention, assisted therapy, treatment design, and various combinations. The databases were Brain, Science Direct, PubMed/Medline, and IEEE. Only papers written in English were considered, and the search was extended to the whole database. Studies were included when (1) systems for upper limb training (uni- and bilateral) were used; (2) systems are based on end-effector and exoskeleton devices (commercially available or not); (3) the clinical intervention was conducted; and (4) the effects of the robot-assisted therapy were investigated.

### 4.2. Robotics in Neurorehabilitation of Upper Limb

According to the Strategic Research Agenda for Robotics in Europe (SPARC) [43], healthcare is seen as a combination of three subdomains: (1) clinical robotics—systems that support care (diagnosis) and cure (surgery) processes; (2) rehabilitation—covering postoperative or postinjury care where direct physical interaction with a robot system will either enhance recovery or act as a replacement for lost function; and (3) assistive robotics—covering other aspects of robotics within the healthcare process where the primary function of the robotic system is to provide assistive help either to carers or directly to patients either in hospital or in a specialist care facility.

Thus, devices to train (robot-aided therapies), support (exoskeletons), or replace (prosthesis) impaired activities or impaired body functions and structures are covered in rehabilitation robotics. In this way, robots are presented as a useful tool in the recovery process in neurological treatment. Such systems participate actively and help the therapist to perform a better rehabilitation process. However, it is not clear in what way and to what extent robotic systems provide this help during the rehabilitation cycle. To improve the quality of help provided, it must be identified how and when the aid is administered.

The summary presented in Table 1 collects the information obtained from the study of several robot-aided neurorehabilitation systems for the upper extremities. The systems selected have been used in clinical trials with patients suffering motor function problems derived from different neurological disorders. A comprehensive reading has been made to identify how robotic assistance has been used, how it has contributed and in which phases of the rehabilitation process. Thus, the present review identifies what the robotic system contributes to the rehabilitation cycle in a quantitative way (measurements), the way it does it (automatic or not), and the phase in which it participates (assessment, assignment, or intervention). Notice that the same robotic systems could cover several phases of the rehabilitation cycle. The more phases are covered, the more automated will be the rehabilitation process.

Rehabilitation, like many aspects of human behaviour, can be thought of as a purposive problem-solving activity [44]. The following review draws upon the problem-solving process from a patient-centred perspective in neurorehabilitation.

#### 4.2.1. Assessment Approaches

As previously indicated, the starting and ending component of the rehabilitation cycle is the functional assessment. It is important to take into account that most of the assessments performed by robotic systems are not functional assessments (carried out in baseline and follow-up stages of treatment), and its provided outcomes are indicators of a patient's performance. Currently, functional assessment is still carried out by traditional tests and scales provided by therapists. The main features of the robot-aided systems reviewed related to the assessment phase of the rehabilitation cycle are described as follows:

*(1) Assessment Mode*. Assessment of the patient's performance can be carried out in two modes: automatic or nonautomatic. The automatic mode corresponds with the online data analysis, that is, during the development or at the end of the session. On the contrary, the nonautomatic mode corresponds with the offline data analysis (after of the end of the session).

*(2) Assessment Method*. Robotic rehabilitation systems present evaluation methods that are based on the biomechanical data they are able to acquire. Based on such data, a rapid report that could be performed in an online or offline mode is provided to the therapist. 74% of the reviewed systems have not specified assessment methods, but propose an evaluation method based on the offline analysis of the biomechanical data acquired during therapy. In these studies, a later analysis of the stored information is done, applying algorithms to obtain information on the patient's performance. However, besides having an automatic record of information, only 26% of the systems perform online processing of these parameters by using specific software (e.g., INMOTION, IPAM, AMADEO, ARMEO, and T-WREX).

*(3) Provided Outcome*. Robot-assisted systems have the advantage of providing a reliable and objective quantitative rapid assessment, based on the comparison of the metrics acquired during therapy. However, this assessment is at the level of impairment but does not provide information on how such impairment influences the activities of the patient's daily life. The most automated are commercially available systems like INMOTION ROBOTS, ARMEOSPRING, AMADEO, REOGO, and DIEGO. They have an online processing that generates a report at the end of the therapy session. However, the reliability of these automatic assessments, although they are based on objective measures, has not been validated with respect to determining, on their own, whether the rehabilitation has been adequate or not. Also, robot-mediated measurements have even smaller dissemination. For this reason, most of the systems reviewed carry out additional clinical evaluation, using functionality scales that are of standardized use at the clinical level, such as those mentioned in Section 3.1, which are still the “gold standard” for measuring outcomes. The interpretation of these scales allows the therapist to determine in an objective way the health condition of the patient and the effectiveness of the treatment.

*(4) Functional Assessment*. Given the importance of making a correct evaluation, it is necessary to highlight the need to use standardized tools and procedures. The classification of the ICF is very useful for this functional assessment. The use of these standard functional scales as the main output of the rehabilitation systems would provide a better and more collaborative way to determine the effectiveness of the therapy based on the metrics obtained by the rehabilitation systems themselves. Currently, this issue is addressed by INMOTION software (INMOTION EVAL) that, based on multiple regression models, calculates Fugl-Meyer Assessment (FMA), Motor Status Score (MSS), Motor Power (MP), and Modified Ashworth Scale (MAS) from the robot-based metrics. These measurements of motor control are highly correlated with the traditional scales [45].

#### 4.2.2. Clinical Decision Support

As previously mentioned, it is important to emphasize that the complexity of a neurorehabilitation treatment usually requires the participation of a work team. Therefore, it is important that the patient's progress information is available to the entire work team, according to the interdisciplinary model. The management of information is one of the more time-consuming tasks that facilitate the decision-making of the therapist. Currently, there are several electronic medical record (EMR) software for the management of the patient's data [46], including based on artificial intelligence [47]. Thus, one of the important aids incorporated in robot-assisted systems is the administration and storage of data automatically, which allows the generation of updated monitoring reports.

The results of the review show that 45% of the systems (the commercial ones) also provide some kind of help in the elaboration of the therapy. The most common assistance is through offering a set of exercises, games (REOGO, DIEGO, and ARMEO), or therapy protocols (INMOTION system) that can be configured or combined by the therapist. One of the systems (REHAROB) also allows the option of selecting exercises that are based on the intervention methods most used in physical rehabilitation, such as the Bobath or Kabat method. On the other hand, in-depth analysis of the data recorded robot-aided therapy, as well as allowing rapid functional assessment, serves as a tool for decision support to determine the patient's discharge. The INMOTION system allows discharge plots to be generated based on the performance of 5 tests that register kinematics and kinetics data. To the authors' knowledge, there are no commercial systems able to automatically generate a complete rehabilitation strategy from the initial functional assessment data and thus the therapist still has to properly identify the patient's problems by means of a reliable diagnosis and the right choice of clinical measures to evaluate the effectiveness of the treatment.

#### 4.2.3. Rehabilitation Approaches and Outcomes

Typically, rehabilitation occurs for a specific period of time but can involve single or multiple interventions delivered by an individual or a team of rehabilitation workers and can be needed from the acute or initial phase immediately following recognition of a health condition through postacute and maintenance phases. Rehabilitation reduces the impact of a broad range of health conditions. Further, neurorehabilitation is often still based on therapists' expertise, with competition among different schools of thought, generating substantial uncertainty about what exactly a neurorehabilitation robot should do [48].

Robot-aided systems allow the training of an impaired limb in multiple sessions and in a systematic way, without loss of efficiency. With respect to the target region of treatment, the number of joints that the same system is capable of treating has been identified. No devices covering the movement of all joints of the upper limb have been found, that is, the shoulder, elbow, wrist, and hand (including fingers joints). The ARMEOSPRING, INMOTION, and ARMEOPOWER systems manage to cover the shoulder and elbow joints and also to train the flexoextension of the wrist and the manual grip, excepting finger joints.

The effectiveness of treatments based on task-specific training in robot-assisted interventions is demonstrated. So it is understandable that 86% of the review systems consider this approach. It is observed that the systems have more than one operating mode (passive, active, active-assisted, or active resistance). This represents a great advantage when considering treatment measures in a flexible way and better adapted to the type of injury. Some systems describe the mechanisms of action of the robots, which can offer assistance to the movement or gravity compensation through cable-based transmissions or pneumatic actuator systems. The pneumatic actuator systems offer the advantage of producing large forces with low weight added to the device, while cable transmission systems have greater shock absorption, smoothness in movement, and greater versatility in their passage through the joints.

Finally, all the robotics rehabilitation systems reviewed are able to acquire and automatically store biomechanical metrics during the therapy. Depending on each robotic system, it can measure the workspace, joint movement ranges, and force exerted, as well as the quality in terms of the precision and smoothness of the trajectories. Other measures derived from the previous ones for a certain interval of time are the speed of execution and completion of the tasks, as well as the reaction times. The acquisition and storage of these parameters are immediate due to the inherent sensorization of the robotic systems (encoders, force sensors, current sensors, etc.). These are objective records due to the robotic intrinsic sensory systems.

## 5. Towards Autonomous Rehabilitation Processes?

The development of autonomous systems is an active line in robotics in general, and with increasing presence in healthcare applications, it is already generating beneficial results as it has done in industry [49]. That is the case of surgical robots in minimally invasive procedures for executing autonomously simple surgical tasks, based on the accuracy of robot movements, image processing algorithms, and cognitive systems. There are many other examples than surgical robotics of translational research applied to healthcare.

The common understanding in the robotic community is that the goal of robotic rehabilitation devices should be to assist therapists in performing the types of activities and exercises they believe give their patients the best chance of a functional recovery. But several barriers have been identified, for the particular case of rehabilitation robotics. The first identified barrier is the lack of effective communication in the planning stage of designing robotics aids, between engineers and therapists. Second, many of the devices are incredibly complicated, from both an engineering and a usability point of view. In fact, “simple-to-use” devices are more likely to be adopted by the clinical community than those that have long set-up times or require multiple therapists and/or aids to use [50]. Another well-known barrier relates to the cost and availability, its relation to the effectiveness of the treatment, and how long the robotic treatment must be applied. Many works discuss these issues. Recent examples are those by Acosta et al., who show that while video games can provide a motivational interface, they are the most effective if designed to target specific impairments [51]. Burgar et al. highlight the importance of providing higher therapy intensities (hours of therapy per day) in an acute stroke study using the MIME robot [52]. Telemedicine and telerehabilitation are promising topics for building remote monitoring and easy to use rehabilitation systems that could allow the work of therapist with patients at home. Serious games and low-cost sensory devices are arising as very promising tools for breaking this barrier. The last barrier, but not the least from the authors' point of view, is the lack of automation, which greatly increases the total cost of the treatments. There is a huge potential to automate the treatment process.

To apply this automation approach to the rehabilitation process, it is first necessary to identify how the process is developed and identify which are the most susceptible elements to be automated, as well as the requirements and limitations to achieve this purpose.

**Figure 2:**
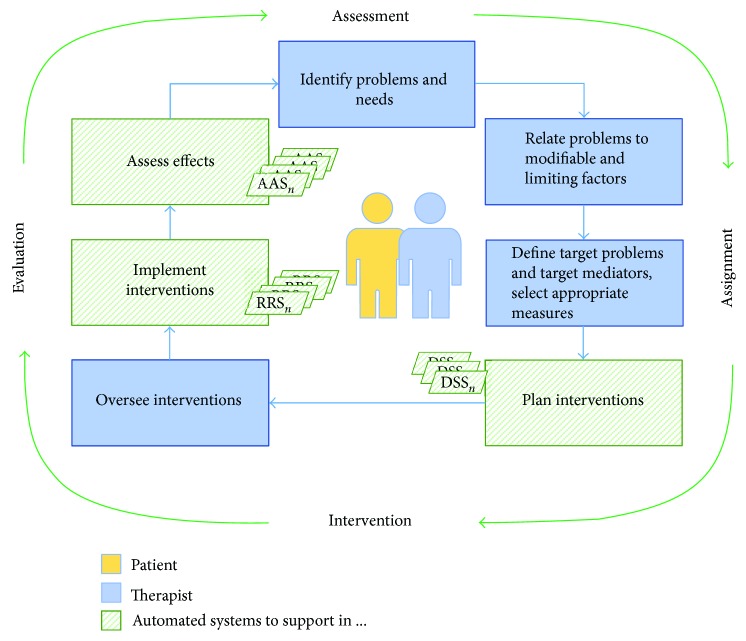
The automated rehabilitation cycle.

Based on the review presented in this article, we have identified three main areas within the rehabilitation cycle where robotics is contributing to automation: planning treatment protocols, implementing interventions, and evaluating the treatment's effectiveness. This rehabilitation cycle, shown in the previous Figure 1, is being transformed into a more automated cycle as shown in Figure 2. This transformation adds more detail but does not alter the rehabilitation cycle, thus maintaining the philosophy centred on the user. In this figure, the main actors (patient and therapist) are supported by several automated tools, as it will be explained below.

### 5.1. The Automated Rehabilitation Cycle

This paper proposes a framework for the development of the rehabilitation cycle that clearly identifies which parts of the process are more likely to be automated, as well as the actors and elements involved. The autonomous rehabilitation cycle would be composed in this way by five elements that are directly correlated with the blocks of the original cycle. According to this approach, three main actors have been identified: user, clinician (understood as the team), and automated systems. Although several automated systems could be available, as denoted in Figure 2, to simplify, we assume that the one used is the best fitted to each case. The appropriate collaboration between the therapy work team and the automated systems is essential to obtain an effective patient-centred rehabilitation process.

**Figure 3:**
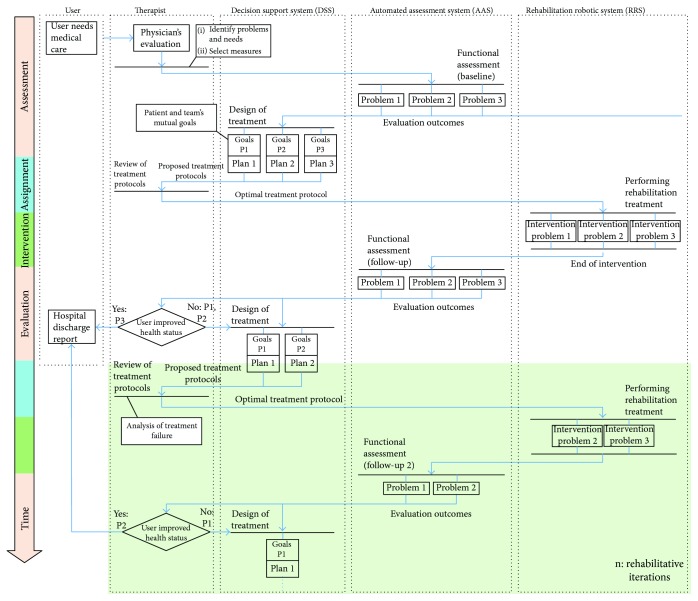
Activity diagram for the automation of the rehabilitation process. In the example shown, three functionality problems were identified in the assessment phase: problem 3 is solved after the first intervention, but problems 1 and 2 remain. Then, the rehab cycle is repeated for *n* iterations.

The interaction between these three participants during the course of an automated neurological rehabilitation process will be described in Figure 3. First, an initial evaluation (interview and exploration-based) is carried out by the clinician to identify the patient's problems and needs and select the most appropriate treatment measures. Also, the appropriate scales for functional assessment are chosen to quantify the level of functionality impairment caused by the neurological injury. Here, where the first automated system is, the automatic assessment system (AAS) performs the functionality assessment using the same clinically accepted scales. The results obtained with the AAS are automatically updated in the patient's clinical history. In addition, these results serve as input parameters to the second automatic system, the decision support system (DSS). The DSS aims at designing the most optimal treatment protocol for the patient, generating the specific intervention plans. This figure is based on the lacks identified in the literature review previously presented.

The therapist discusses with the patient to review and adjust the objectives, deciding which treatment plans proposed by the DSS will be adopted. Then, the selected robotic rehabilitation systems (RRS) perform the intervention. After the intervention with the RRS, an assessment of functionality similar to the initial one is carried out again, in order to quantify the effectiveness of the therapeutic measures. For this, the AAS is used again. Finally, if all the problems identified are considered resolved or accepted by both the clinician and the patient, the rehab cycle is concluded. Otherwise, the necessary iterations will be made to try to solve the remaining problems.

It can be deduced that the proposed automated systems operate separately and independently but that they are intrinsically connected and depend on each other for efficient operation, in coordination with the clinician and the patient. The methods to extract metrics and share them and their degree of acceptance by both users and health professionals should be rationalized and assessed, as a prerogative to achieve the automation. To design assistance rehabilitation systems, although the focus is on the subject to be treated, it is important to systematize the understanding of the requirements demanded by therapists in order to enable an easier integration of technology in their daily activities [53].

By providing low-cost and easy to access tools for implementing this automated rehabilitation cycle, the viability of extending the rehabilitation cycle can be increased, not only as a temporary activity but also as a lifelong rehabilitation, as needed, for example, for affordable robotic therapy in maintaining function in degenerative disorders.

Thus, in the opinion of the authors, the requirements that the components of a rehabilitation cycle must meet to be more autonomous are described below.

#### 5.1.1. Automated Assessment Systems (AAS)

As revealed by the analysis of assessment methods in neurorehabilitation, the use of traditional motor and functional scales is the main approach to determine the effectiveness of the rehabilitation process. For this reason, the development of methods based on traditional assessment scales that are widely used and known by specialists in rehabilitation is one of the lines of research that have been highlighted to achieve a more autonomous rehabilitation cycle.

There are already oriented studies in this line of work, taking into account two premises: the method and metrics. Regarding the method, tests that are administered without direct contact of the professional are more suitable to be automated. Concerning metrics, it is essential to assess which ones give relevant information and are less invasive for the subject to be evaluated [54].

It can be seen that the FMA is one of the most used scales employed for the motor assessment in the clinical trials that this review included. So it appears reasonable that the potential for the automation of these kinds of assessment methods is being studied. The application of RGB-D sensors, inertial measurement sensors, and other sensors has allowed the scoring of a part of the FMA to be automated [55]. However, one of the biggest problems with the evaluation using traditional tests is the time they take the therapist to administer. Other works address automatic administration of assessment procedures, such as the case of BBT [56]. Even so, a large number of scales and the variety of methods (sensor-based, tracking systems, computer-based, etc.) make the topic of automating the assessment a very promising line of research.

In this respect, the literature also presents several projects that are focused on the automation of the traditional and still “gold standard” scales. As traditional scales are widely used in clinical trials in rehabilitation, as seen in this article, and because the administration of the evaluation is time-consuming, it appears reasonable that the automation of these kinds of assessment methods is being studied. There is an important difference in emphasis between clinical assessment and measurement. Traditional scales comprise several items. However, measurement concerns the quantification of an attribute and some studies [57] demonstrate that multi-item measures need only a few carefully chosen items to generate reliable and valid estimates.

Following the model of the rehabilitation process, most of the systems reviewed (based on end-effector or exoskeletons) are clearly located within the intervention stages of the rehabilitation cycle. However, a percentage of them (46% end-effector and 43% exoskeletons) addresses the assessment stage, based on the metrics that are obtained from the use of systems in therapy. This assessment serves as a method of “rapid assessment” to support the therapist and inform the patient of the effectiveness of the rehabilitation process, but there are few works that report comparative studies or clinical trials to validate nonclinical metrics.

#### 5.1.2. Decision Support System (DSS)

Decision support systems based on artificial intelligence (AI-powered DSS) are one of the most active fields in recent years, and it is expected that they will soon contribute to the decision-making process. In healthcare, a variety of software for EMR management is already available (see Section 4.2.2) to help the therapist in decision-making. However, the diagnosis of diseases still presents serious limitations. We can find numerous smartphone apps that allow an online diagnosis, yet the reliability of the diagnosis is not yet consistent with that of a doctor [58]. Besides, researchers in the artificial intelligence community have started to design robot-assisted rehabilitation devices that implement artificial intelligence methods to improve upon the active assistance techniques found in Section 4.2.3.

Clinical decisions are an important component of the rehabilitation cycle, since they involve the determination of the objectives and design of the rehabilitation treatment. As can be seen in this review, the support provided by automated systems for this kind of task is by providing more reliable and objective information about the motor performance of the user during the intervention, as well as allowing the execution of different types of intervention procedures that can be configured by the clinician.

Regarding the assignment stage of the rehabilitation cycle, there are two steps that could be automated by using artificial intelligence techniques: the planning of intervention treatments and the assignment of the appropriate RRS for intervention.

Related to the planning of intervention treatments, the generation of these protocols is based on different factors that depend on the type of lesion and on how it affects the development of the patient's daily living activities. Many of the intervention measures are systematized in order to deal with a particular effect (concrete measures for specific problems), but there is no reason to believe that a “one-size-fits-all” optimal treatment exists. Instead, therapy should be tailored (intensity, number of repetitions, and duration of the intervention) to each patient's needs and abilities [59]. In addition, the protocol planning should consider the available tools (RRS) to execute such protocol in order to assign the appropriate RRS to the type of lesion (e.g., a hand injury cannot be trained by a device designed for elbow training).

Thus, we have identified some requirements that must be met to develop intelligent systems for treatment planning: (1) coherence between technological and traditional outcome measures, for the purpose of a therapeutic intervention based on technology and the problem-solving approach; (2) differentiating these measures according to the level of the effect (mild, moderate, and severe); (3), based on models, to identify the parameters that define an adequate physical condition according to the demographics of the patient and healthy profiles; (4) to be able to estimate the physical condition of the user to compare it with the welfare reference model; and (5) to generate a protocol that can be executed by the available intervention systems.

These requirements imply that the integration of an AI-powered DSS in the automated cycle requires as input parameters the results of the evaluation systems (AAS) and, based on them, generates an optimized treatment protocol that can be executed by the systems of automatic intervention (RRS). This is why special attention is needed to the development of strategies that allow the integration and collaborative execution of these automated systems.

#### 5.1.3. Robotic Rehabilitation Systems (RRS)

The developments in medical robotics systems and RRS are fields that have awakened most interest for research in robotics. Due to the direct participation in the intervention phase, the different methods used in rehabilitation (task-oriented, constraint-induced, etc.), and the understanding of what constitutes the most, appropriate therapy has the potential to become an intensively active topic of research [59].

Two main issues have been highlighted: the ability of the RRS to acquire multiple information on patient performance during the development and the fact that from these data an assessment of patient functionality is obtained, even in the same type of score as the traditional scales.

However, the type and amount of information that is obtained depend a lot on the type of robotic system (end-effector or exoskeleton) and the intrinsic sensory system. Also, the parameters derived from the measurements, as indicators of quality (accuracy, smoothness, etc.), can be very heterogeneous. Therefore, a critical issue is to unify the metrics acquired by the RRS, so that they provide as much information as possible for a rapid assessment by the therapist and not just raw data. Thus, among this type of metrics we have the following: range of movement, speed, precision, efficiency, percentage of work of the patient and percentage of work of the robot, and degree of attention in the task. All the works reviewed coincide in capturing the kinematic data; however, they do not address high-level indicators such as the percentages of robot and patient work (excepting NeReBot that gives it as a percentage) nor the degree of attention.

Another important issue is to promote the adherence of the user to therapy. It is necessary to provide an adequate feedback that motivates the patient. Using virtual reality systems is the most widely used solution for this purpose. However, it is important not only the way in which the feedback is given but also the information provided to the user. In this sense, therapists agree that a visual feedback that tells the user if he has improved his score during the execution of the therapy would be beneficial. Other high-level indicators such as the percentages of robot and patient work, control signal, or kinematic data could be helpful to the user only if they help to show the relevance of the patient's progress.

RRS-type systems are already integrated into the rehabilitation cycle, due to their imminent nature in the intervention; however, addressing the aforementioned questions would allow the rest of the automated components indicated in this paper (AAS and DSS) to take advantage of the objective information that is acquired with the RRS.

## 6. Conclusions

A new automated rehabilitation framework has been proposed based on a literature review of robotic rehabilitation systems (RRS) for the upper limb treatment, highlighting its relation with the rehabilitation cycle. This framework has been presented regarding the implementation of more autonomous rehabilitation procedures. Three automated elements were described to make up the proposed framework: automated assessment systems (AAS), decision support systems (DSS), and robotic rehabilitation systems (RRS).

The development of AAS should be based on the traditional assessment methods, since the traditional scales are still the “gold standard” for measuring outcomes and determine the effectiveness of treatment. In addition, the outcome provided by the AAS is obtained in an objective way, generating additional information about the user's performance.

Those systems must be complemented with a novel DSS to help in clinical decision-making and treatment planning. The management of the patient's data (EMR) is currently addressed by using specific software based on high-level algorithms and also on artificial intelligence (AI). Optimized treatment protocols customized to the patient's condition are expected to be automatically generated by these DSS. For this purpose, AI is a promising tool. Dealing with multiple objectives in decision-theoretic planning and reinforcement learning algorithms [60] could contribute to allow the optimal protocols to be generated. Thus, the treatment protocols could require only approval or adjustment by the clinician.

To conclude, the implementation of the proposed framework should consider some issues that are summarized as follows:

- The development of strategies for allowing the integration and collaborative execution of these automated systems is needed. It must be considered a proper data management in order to allow the AAS and DSS to use the objective information that is acquired with the RRS. In this way, a communication channel similar to the interdisciplinary team model will be enabled for the automated elements.
- In the case of the AAS development, the automatic administration of the assessment must be considered and not only the automation of the outcome. Knowledge of the user is as important as system functionality, since without the user's cooperation and acceptance, the system's functionality may be ineffective.
- The complexity of neurological disorders and its effect normally presents additional diseases concurrent with the primary disorder (comorbidity) that could limit the patient recovery.
- The feasibility of using AI to generate optimal treatment protocols is still unclear, but considering that AI is a mature science at present, the potential to contribute to the implementation of the proposed DSS is encouraging.
- Clinical protocols are validated through randomized control trials (RCT) where a large number of patients undergo the same treatment. In this regard, the most homogeneous samples must be recruited for RCTs that is challenging because of the inherent nature of neurological disorders.

Robots are currently viewed as advanced therapy tools under a therapist's guidance. However, the implementation of the above-mentioned systems could lead to more autonomous and intelligent processes in neurorehabilitation.

## Figures and Tables

**Table 1:** Review of robot-aided system for upper limb neurorehabilitation.

System	Assessment				Assignment		Intervention				
	Automatic assessment	Assessment method	Provided outcome	Functional assessment	Therapy planning support	Method	Rehabilitation target	Task-specific training	Method	Interaction	Measurements
*End-effector-type system*											
ACT-3D [61]	No	Offline data analysis	Difference between sessions	FMA	No	N/A	Shoulder; elbow	Yes	Reach (payload simulation)	VR; haptic; auditory	Kinematic data; force
ARM GUIDE [62, 63]	No	Offline data analysis	Difference between sessions	CM; RLAFT	No	N/A	Shoulder; elbow	Yes	Passive; active assisted; active resistance	VR; haptic	Kinematic data; force; straightness; smoothness
BRACCIO DI FERRO [64, 65]	No	Offline data analysis	Difference between sessions	FMA; MAS	No	N/A	Shoulder; elbow	Yes	Active assisted; active resistance; gravity compensation	VR; haptic	Kinematic data; force; smoothness; accuracy
GENTLE/S [66, 67]	No	Offline data analysis	Visual comparison of data trends (slopes)	FMA; MoAS; MAS; NSA; SCT	Yes	Haptic Master software allows the therapist to define the exercise path	Shoulder; elbow	Yes	Passive; assisted; active; trajectory correction	VR; haptic	Kinematic data; force
INMOTION ARM [59, 68]	Yes	INMOTION EVAL software; 5 evaluation tests, robot generates 4 evaluation reports	Robot calculates 13 evidence-based measures of motor control that are highly correlated with traditional scales	FIM; FMA; MP; NIHSS	Yes	Selection of the therapeutic exercise games; progress measurement of determined medical necessity; assesses treatment based upon measurable gains	Shoulder; elbow	Yes	Passive; active assisted; active resistance; gravity compensation	VR; haptic	Kinematic and kinetic data (position, direction, distance, area, time, force, smoothness, accuracy); performance measures
IPAM [69, 70]	Yes	IPAM software	Virtual environment feedback	FMA	Yes	Automatically generated exercises based on a clinical assessment of the patient (automated tasks)	Shoulder; elbow	Yes	Passive; active resistance; active; reach	VR; haptic	Kinematic data; force
MEMOS [71, 72]	Yes	Online data analysis	Score proportional to the voluntary motor activity developed during the task	FMA; MSS; MRC; MP	No	N/A	ARM: not a specific joint	Yes	Passive; active resistance; active	VR; haptic	Kinematic data; force
MINE [73]	No	Offline data analysis	Difference between sessions	FMA; MSS; BI; FIM; MP; MAS	No	N/A	Shoulder; elbow	Yes	Passive; active assisted; active resistance; bilateral	VR; haptic	Kinematic data; force [bimanual]
NEREBOT [74, 75]	Yes	Online data analysis	Feedback based on patient's effort	FMA; MRC; FIM; MAS; FAT; BBT	No	Therapist defines the exercise by choosing via points and adjusts robot parameters according to the type of exercise	Shoulder; elbow; forearm	No	Passive; active assisted; active	VR; auditory	Kinematic data; patient effort (cable tension based)
REHAROB [76, 77]	Yes	Online data analysis	Feedback based on patient's effort	FMA; FIM; MAS; RMA; BI; BMR	Yes	Therapist can choose exercises from any therapeutic school (Bobath, Kabat, etc.)	Shoulder; elbow; forearm	No	Passive	N/A	Kinematic data
AMADEO TYROMOTION [78–80]	Yes	TyroS software	Difference between sessions	FMA; MRC; MI; MAS; FIM; COPM	Yes	TyroS software creates a therapy report and therapy progress	Hand: prehension	Yes	Passive; active assisted; active	VR	Kinematic data; force; tonus; spasticity
BIMANUTRACK [81, 82]	No	Offline data analysis	Difference between sessions	FMA; WMFT; RMA; MAS	Yes	Programming of individually adjusted natural gait trajectories; real-timesimulation of the programmed foot trajectory	Forearm; wrist	No	Active; passive [bilateral]	N/A	Kinematic data; force [bimanual]
HWARD [83, 84]	No	Offline data analysis	Difference between sessions	ARAT; BBT; FMA; NIHSS; GDS; NSA; 9-HPT;SIS; MAS	No	N/A	Wrist; hand	Yes	Passive; active assisted; active resistance	VR	Kinematic data; force
REOGO [85, 86]	Yes	Advanced management software	Evolution of measurements	FMA; MFT	Yes	Library with a wide range of engaging exercises and games for various rehabilitation objectives	Shoulder; elbow; wrist; hand	Yes	Passive; active; guided; free	VR; haptic	Kinematic data; accuracy; smoothness; force; muscle tone
DIEGO [78, 87]	Yes	TyroS software	Evolution of measurement	N/A	Yes	Selection of therapy games	ARM: not a specific joint; shoulder	Yes	Passive; assistive; active; gravity compensation [uni- and bilateral]	VR; haptic	Kinematic data; motoric function; force proprioception
*Exoskeleton/Orthosis system*											
L-EXOSPERCRO [88]	No	Offline data analysis	Difference between sessions	FMA; MAS; BAS	Yes	Selection of different trajectories in the same virtual environment	Shoulder; elbow	Yes	Active; active assisted; gravity compensation	VR	Kinematic data; force; accuracy
MYOPRO [89–91]	No	EMG signals	Difference between sessions	MAS; BBT; FMA; MAL-AOU;MAL-HW	No	N/A	Elbow	Yes	Active assisted	Haptic	Kinematic data; force
WREX [92]	No	Offline data analysis	Difference between sessions	N/A	No	N/A	Shoulder; elbow	Yes	Active assisted; gravity compensation	N/A	Kinematic data; force
ARMEOSPRING (T-WREX) [93–95]	Yes	Java Therapy 2.0 software	Difference between sessions	FMA; RFT: BBT: BBTD	Yes	Selection of therapy games	Shoulder; elbow; forearm	Yes	Passive; gravity compensation	VR; haptic	Kinematic data; force
MENTOR PRO (hand) [96–98]	No	Offline data analysis	Monitoring of patient's progress in DLA	N/A	No	N/A	Wrist; hand; fingers	No	Active (only extension)	VR	Kinematic data
HEXORR [99]	No	Offline data analysis	Difference between sessions	ARAT; FMA; MAS	No	N/A	Metacarpus [interfalangica]	Yes	Passive; active assisted; active; gravity compensation	Haptic	Kinematic data; force grasping patterns
RUTGERS MARTER II [100, 101]	Yes	Online data analysis	Performance meter	JTHF; FMA	No	N/A	Hand	Yes	N/A	VR; haptic; auditory	Kinematic data; force
SUPINATOR-EXTENDER [102]	No	Offline data analysis	Difference between sessions	N/A	No	N/A	Forearm; wrist	Yes	Active assisted	VR; haptic	Kinematic data; force; grasping patterns
T-WREX [95, 103]	Yes	Java Therapy software	Monitoring of a patient's progress by skilled rehabilitation therapist	FMA; RFT; BBT	No	N/A	Shoulder; elbow; forearm; wrist; hand	Yes	Passive; gravity compensation	VR	Kinematic data; force
WOTAS [104]	No	Offline data analysis	Difference between sessions	FTMTRS	No	N/A	Elbow; forearm; wrist	Yes	Tremor control	Haptic	Kinematic data (voltage coming from gyroscopes)
ARMEOPOWER (ARM-IN III) [93, 105, 106]	Yes	GIANTS game engine	Difference between sessions	FMA	Yes	Choose from several VR therapy tasks	Shoulder; elbow; forearm; wrist; hand	Yes	Active; passive; active assisted; active resistance; gravity compensation	VR	Kinematic data; force
ARM-IN [107, 108]	Yes	Online data analysis	Difference between sessions	N/A	Yes	Allows different therapy modes, robot interface for patient (PRI) and therapist (TRI)	Shoulder; elbow; forearm; wrist	Yes	Passive; active assisted; active	VR; haptic	Kinematic data; force
GENTLE/G [109]	Yes	Online data analysis	Performance meter	N/A	Yes	HMI module for interaction between the user and the Gentle robot; database for storing patient information	Shoulder; elbow; hand	Yes	Passive; assisted; active; trajectory correction	VR; haptic	Kinematic data (position, direction, distance); force
Rupert [110, 111]	No	Offline data analysis	Difference between sessions	WMFT; FMA	No	N/A	Shoulder; elbow; forearm; wrist	Yes	Passive; active assisted; active	VR; haptic	Kinematic data; force; [motor activity; stroke impact scale; stroke recovery scale]

9-HPT: 9-Hole Peg Test; ARAT: Action Research Arm Test; BAS: Bimanual Activity Scale; BBT: Box and Blocks Test; BBTD: BBT (without picking up blocks); BI: Barthel Index; BMR: British Medical Research; CM: Chedoke-McMaster; COPM: Canadian Occupational Performance Measure; FAT: Frenchay Arm Test; FIM: Functional Independence Measure; FMA: Fugl-Meyer Assessment; FTMTRS: Fahn-Tolosa-Marin Tremor Rating Scale; GDS: Geriatric Depression Scale; JTHF: Jebsen Test of Hand Function; MAL-AOU: Activity Log-Amount of Use; MAL-HW: Motor Activity Log-How Well; MAS: Modified Ashworth Scale; MFT: Manual Functional Test; MI: Motricity Index; MoAS: Motor Assessment Scale; MP: Motor Power; MRC: Medical Research Council; MSS: Motor Status Score.
